# Predicting progression of mild cognitive impairment to dementia using neuropsychological data: a supervised learning approach using time windows

**DOI:** 10.1186/s12911-017-0497-2

**Published:** 2017-07-19

**Authors:** Telma Pereira, Luís Lemos, Sandra Cardoso, Dina Silva, Ana Rodrigues, Isabel Santana, Alexandre de Mendonça, Manuela Guerreiro, Sara C. Madeira

**Affiliations:** 1 Instituto Superior Técnico, Universidade de Lisboa, Lisbon, Portugal; 2 INESC-ID, R. Alves Redol 9, 1000–029 Lisbon, Portugal; 3 Cognitive Neuroscience Research Group, Department of Psychology and Educational Sciences and Centre for Biomedical Research (CBMR), University of Algarve, Algarve, Portugal; 4 Faculdade de Medicina, Universidade de Coimbra, Coimbra, Portugal; 5 Departamento de Neurologia, Centro Hospitalar e Universitário de Coimbra, Coimbra, Portugal; 6 LASIGE, Faculdade de Ciências, Universidade de Lisboa, R. Ernesto de Vasconcelos, 1749–016 Lisbon, Portugal

**Keywords:** Neurodegenerative diseases, Mild cognitive impairment, Prognostic prediction, Time windows, Supervised learning, Neuropsychological data

## Abstract

**Background:**

Predicting progression from a stage of Mild Cognitive Impairment to dementia is a major pursuit in current research. It is broadly accepted that cognition declines with a continuum between MCI and dementia. As such, cohorts of MCI patients are usually heterogeneous, containing patients at different stages of the neurodegenerative process. This hampers the prognostic task. Nevertheless, when learning prognostic models, most studies use the entire cohort of MCI patients regardless of their disease stages. In this paper, we propose a Time Windows approach to predict conversion to dementia, learning with patients stratified using time windows, thus fine-tuning the prognosis regarding the time to conversion.

**Methods:**

In the proposed Time Windows approach, we grouped patients based on the clinical information of whether they converted (converter MCI) or remained MCI (stable MCI) within a specific time window. We tested time windows of 2, 3, 4 and 5 years. We developed a prognostic model for each time window using clinical and neuropsychological data and compared this approach with the commonly used in the literature, where all patients are used to learn the models, named as First Last approach. This enables to move from the traditional question “Will a MCI patient convert to dementia somewhere in the future” to the question “Will a MCI patient convert to dementia in a specific time window”.

**Results:**

The proposed Time Windows approach outperformed the First Last approach. The results showed that we can predict conversion to dementia as early as 5 years before the event with an AUC of 0.88 in the cross-validation set and 0.76 in an independent validation set.

**Conclusions:**

Prognostic models using time windows have higher performance when predicting progression from MCI to dementia, when compared to the prognostic approach commonly used in the literature. Furthermore, the proposed Time Windows approach is more relevant from a clinical point of view, predicting conversion within a temporal interval rather than sometime in the future and allowing clinicians to timely adjust treatments and clinical appointments.

**Electronic supplementary material:**

The online version of this article (doi:10.1186/s12911-017-0497-2) contains supplementary material, which is available to authorized users.

## Background

Decline in cognitive functions, together with other evidences of neurological degeneration, become increasingly likely as people age (some at an earlier age others at a faster rate) [1]. Therefore, distinguishing normal aging from cognitive decline due to pathological processes and understanding the individualized disease diagnostic and prognostic patterns are ongoing research challenges. Neurodegenerative diseases causing cognitive impairment, such as Alzheimer’s disease (AD) and other forms of dementia (dementia with Lewy Bodies (DLB), frontotemporal dementia (FTD), Vascular dementia (VaD)) are amongst the best studied diseases of the central nervous system due to its devastating effects on patients and their families, and to the socio-economic impact in modern societies [2]. Nowadays, over 46 million people live with dementia (mostly AD) worldwide and this number is estimated to increase to 131.5 million by 2050 [2]. Unfortunately, by the time patients meet criteria for dementia, the brain has suffered sufficient damage to severely impact cognition and autonomy. With this in mind, recognizing putative progress to dementia when patients experience only mild cognitive deficits, at a stage of Mild Cognitive Impairment (MCI), is paramount to develop disease-modifying therapies and identifying appropriate therapeutic windows [3–9]. Clinical studies with MCI patients have reported higher risk rates of conversion to dementia (in particularly to AD) than community studies, suggesting these patients as a group of singular interest to follow-up studies and interventions [10, 11]. In a recent systematic review [12], MCI diagnosis was associated with an annual conversion rate up to 20%, however with substantial variation in risk estimates.

In this context, researchers have followed a number of different directions for prognostic prediction in MCI. Some explored biological markers, such as those in cerebrospinal fluid (CSF) or brain imaging (using magnetic resonance imaging (MRI) or positron emission tomography (PET) technologies) [3, 13–20]. Others used neuropsychological tests (NPTs) alone [8, 10, 11, 21–25] or in combination with biological markers [9, 26–31]. The latter strategy seems to achieve better predictive performances than using the markers independently [3, 9, 15, 30–32]. Despite the efforts, to date, no single biomarker to predict conversion from MCI to dementia with high accuracy was yet found [9].

Furthermore, it is widely recognized that neurodegenerative diseases take many years to manifest, slowly draining the cognitive capabilities of those they afflict. This makes it hard to ascertain where a given MCI patient stands in the continuum of the disease. As such, cohorts of MCI patients are usually very heterogeneous, with patients at different stages of the neurodegenerative process. This patients’ heterogeneity, if not considered, introduces noise in the prognosis methods, decreasing their reliability [16, 31, 33]. To our knowledge few studies take this issue into consideration [33, 34]. Some addressed this question [16, 35] by performing an a posteriori evaluation of the results, looking for differences induced by the conversion time. Doyle et al. [16] developed a continuous index of disease progression based in multivariate ordinal regression and showed that patients considered as “late converters” (converting in a 24–36 months follow-up) were characterized by a different distribution from those that converted within a 12 months follow-up. Adaszewski et al. [35] tested diagnostic accuracy at different points of conversion to AD (4 years before dementia to 2 years of clinical dementia) using Support Vector Machines (SVMs) classification with structural magnetic resonance imaging. However, a heterogeneous cohort of MCI patients is used to learn the model and the emergent differences putatively caused by the time a patient takes to convert are evaluated a posteriori. We name this approach as First Last (FL) approach, as it combines the baseline and the clinical outcome at the last evaluation of each patient when building the learning examples, regardless their time to conversion.

In this work, we propose a Time Windows approach to tackle the MCI-to-AD conversion problem. We used NPTs and the time to conversion of MCI patients is handled during the construction of machine learning examples, where the set of patients is divided into subgroups according to their conversion time and later used by classifiers. As such, unlike other studies, the prognostic model is trained with time-homogeneous MCI groups and thus learns already from putatively different progression patterns of disease. Two precedent works used temporal approaches to study progression to Alzheimer’s disease using neuroimaging data [33, 34]. Different groups of converting MCI patients were created by using scans (from FDG-PTE [33] or MRI [34]) collected at 6 to 36 months before the subjects fulfill the AD criteria. Then, distinct prognostic models were learned for each of those groups and the single group of non-converting MCI patients. Although this case constructs learning examples differently and uses other data types, the results corroborate our hypothesis that prognostic predictions can be improved by learning with subjects at similar stages of the disease. Our approach is different from the already proposed [33, 34] since we stratify both stable and converter MCI patients while in the previous studies only the converting group is homogenized. We note that in this context a stable MCI patient in a time window may become a converter MCI patient in a larger time window as happens in clinical practice. We also emphasize that the follow-up time used in our work is longer (time windows of 4 and 5-years were studied). Furthermore, we tested the Time Windows approach with neuropsychological data, which to our knowledge was not done so far. The reason behind this decision is the fact that we believe it is fundamental to study the predictive power of NPTs, since they are widely used in clinical practice in alternative to more expensive and often invasive approaches and these tests are still a hallmark for diagnosis of dementia and MCI. In fact, the technology required for PET imaging and other biomarkers may not be widely available, while NPTs are routinely used in clinical practice. In addition, current theoretical models suggest that neuropsychological data may be more important in identifying MCI patients who are closer to convert to dementia, while neuroimaging and biological markers may identify the presence of neurodegenerative pathology in subjects that will develop dementia in the future [8, 36]. Moreover, although machine learning approaches are gaining relevance in dementia research [15, 33], studies including only NPTs are mostly based on traditional statistical analysis instead of machine learning.

Another advantage of the proposed approach, learning with homogeneous groups instead of learning with heterogeneous groups as it is widely performed using the FL approach, concerns the relevance of the clinical question addressed. From a clinical standpoint, knowing that a MCI patient will convert to dementia but not knowing if this will happen in the following year or in the next 20 years is not particularly useful. However, knowing that the conversion will occur in a particular time window, for instance within 5 years, is clearly useful. This allows the clinicians to adjust the therapeutics to match the effective progression of the disease and to schedule clinical appointments accordingly.

**Fig. 1:**
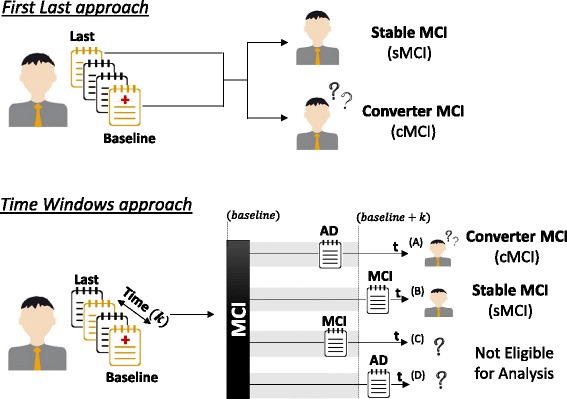
Creation of learning examples following either the First Last approach or the Time Windows approach. A new class is created to define the type of patient’s progression (converting (cMCI) or non-converting (sMCI)) in the interval of k years from the baseline assessment (Time Windows approach) or with no time restrictions (FL approach)

Figure 1 illustrates the problem addressed in this work: using neuropsychological data to predict whether a patient with MCI will convert to dementia using specific time windows (2, 3, 4 and 5 years) and comparing it with the First Last approach, where time windows are not used.

## Methods

**Fig. 2:**
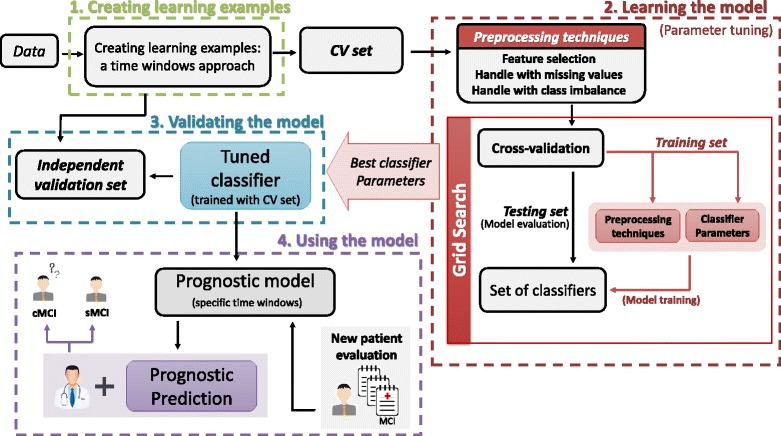
Workflow of the proposed supervised learning approach to predict MCI-to-dementia conversion, based on time windows. It comprises four steps: **1**) Data Preprocessing (construction of learning examples based on time windows), ***2***) Model Learning (tune the model for each time window and FL datasets), ***3***) Model Validation (validate the model (tuned to the CV set) with an independent validation set) and ***4***) Using the model (Prognostic prediction of new MCI patients)

We start by describing the data. Then, we describe each step of the proposed supervised learning approach using learning examples with time windows (illustrated in Fig. 2). This approach comprises four steps, further discussed in the following subsections: 1) Creating learning examples using time windows, 2) Learning the prognostic model, 3) Validating the prognostic model and 4) Using the model.

### Data

Participants were selected from the Cognitive Complaints Cohort (CCC) [23], a prospective study conducted at the Faculty of Medicine of Lisbon to investigate the progression to dementia in subjects with cognitive complaints based on an extensive neuropsychological evaluation at one of the participating institutions (Laboratory of Language Studies, Santa Maria Hospital, and a Memory Clinic, both in Lisbon, and the Neurology Department, University Hospital in Coimbra).

The inclusion criteria for admission to CCC were presence of cognitive complaints and completing assessment with a neuropsychological battery designed to evaluate multiple cognitive domains and validated for the Portuguese population (*Bateria de Lisboa para Avaliação das Demências* – BLAD [37]). The exclusion criteria for the admission to CCC were diagnosis of dementia (according to DSM-IV [38]) or other disorders that may cause cognitive impairment, namely stroke, brain tumor, significant head trauma, epilepsy, psychiatric disorders (such as severe depression), uncontrolled medical illness, sensory deficit or medical treatments interfering with cognitive function, and alcohol or illicit drug abuse. For the purpose of this study, participants were diagnosed with Mild Cognitive Impairment when fulfilling the criteria of the MCI Working Group of the European Consortium on Alzheimer’s disease [39]:

1. Cognitive complaints coming from the patients or their families;
2. Report of decline in cognitive functioning relative to previous abilities during the past year by the patient or informant;
3. Presence of cognitive impairment (1.5 standard deviations below the reference mean) in at least one neuropsychological test;
4. Absence of major repercussions on daily life activities.

At follow-up, participants could also be diagnosed with dementia according to the DSM-IV [38] criteria. The study was conducted in accordance with the Declaration of Helsinki, and was approved by the local ethics committee. Informed consent to participate in the study was obtained from all participants.

**Fig. 3:**
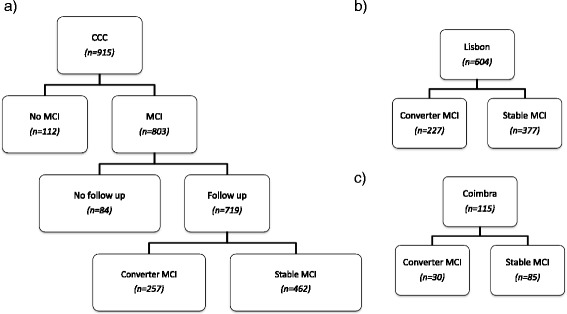
Flow chart of the final number of Cognitive Complaints Cohort (CCC) participants: **a** complete cohort; **b** cohort of patients recruited in Lisbon; **c** cohort of patients recruited in Coimbra

From the CCC cohort of 915 patients, 803 cases fulfilled the criteria for MCI diagnosis at baseline (Fig. 3a). Only patients with follow-up were selected, which was the case for 719 patients, who had mean age (M ± SD) of 69.4 ± 8.5 years, formal education (M ± SD) of 8.2 ± 4.7 years, follow-up (M ± SD) of 3.3 ± 2.8 years and, gender distribution (male/female) of 289/430. 257 (36%) patients converted to dementia (converter MCI) and the remaining 462 (64%) cases did not convert throughout the study (stable MCI). Demographic and clinical characterization data is presented in Table 1. Differences among converting and non-converting MCI patients were assessed by independent samples t-tests for numerical data (age and years of formal education) and by the χ^2 Pearson Chi-Square for nominal data (gender), using IBM SPSS Statistics 24 (released version 24.0.0.0). A *p*-value <0.05 was assumed as statistically significant. The dataset includes 129 variables covering clinical, demographic and neuropsychological data. These variables are further described in appendix by means of two tables: one describes the cognitive domains assessed by each measure and the other reports the mean average and missing values percentage for each feature and group of patients used in this study [See Additional files 1 and 2]. The neuropsychological assessment was standardized according to age and education norms for the Portuguese population and z-scores were calculated.

**Table 1:** Baseline demographic and clinical characterization data

	*Converter MCI* *(n = 257)*	*Stable MCI* *(n = 462)*	*p-value*
Age, years (M ± SD)	71.7 ± 7.8	68.1 ± 8.6	< 10^−8^
Gender (male/female)	93/164	196/266	0.102^*#*^
Formal education, years (M ± SD)	8.9 ± 4.9	8.8 ± 4.7	0.612
Follow-up time, years (M ± SD)	2.9 ± 2.3	3.5 ± 3.0	0.007

Group comparisons (converter MCI vs. stable MCI) were performed with independent samples t-tests (or χ^2 Pearson Chi-Square test when appropriate^#^). Statistically significant (*p* < 0.05)

Since subjects were evaluated by different clinicians in two Portuguese hospitals (in Lisbon and Coimbra) we may distinguish two independent cohorts of patients from this cohort (Fig. 3b and c). For the purpose of the proposed supervised learning approach, the patients recruited in Lisbon (*n* = 604) constitute the cross-validation (CV) set and are used to learn the prognostic prediction model, while the patients recruited in Coimbra (*n* = 115) are subsequently used as an independent validation set to validate the model.

### Supervised learning approach using learning examples created with time windows

The first step of the proposed supervised learning approach consists in creating the learning examples using time windows. Then, the model and parameters are tuned to the CV set under a cross-validation scheme (Learning the model, Fig. 2) and finally validated using an independent validation set (Validating the model, Fig. 2). The model predicts whether a patient diagnosed with MCI at *baseline* converts to dementia (or remains MCI) at time *baseline* + *k*. The time *k* (in years) corresponds to the considered time window. The model may then be used in clinical practice (Using the model, Fig. 2). This process is repeated for each time window and FL datasets. The prognostic prediction approach was implemented in Java using WEKA functionalities (version 3.7.1) [40].

#### Creating learning examples using time windows

The original data must be transformed to create learning examples to be used by supervised learning techniques. A learning example depends on the changes in the patients’ diagnostic between the baseline and a follow-up evaluation (prognosis). It is composed by: 1) the baseline assessment of a MCI patient (first evaluation), and 2) a new attribute representing the type of progression of that patient (conversion or non-conversion), computed using the clinical diagnosis at a follow-up evaluation (usually called class label). This latter evaluation (used to compute the class) is the last evaluation in the FL approach and an evaluation inside the time window in the proposed approach. We note that since we are performing prognostic prediction, NPTs used to learn the model are never used to compute de class. We propose a new strategy to create learning examples using time to conversion to pool patients in similar stages of disease (termed Time Windows approach). Figure 1 illustrates the creation of learning examples using the Time Windows approach in comparison with the widely used in the literature, the First Last approach. Follows a description of learning example creation for these approaches.

(1) *First Last approach:* Combines the baseline with the last evaluation of each patient. If the patient was diagnosed as MCI at the last evaluation, a learning example labelled as stable MCI (sMCI) is created. If the final diagnostic is dementia the learning example is labelled as converter MCI (cMCI). The features (clinical and neuropsychological measures) are taken from the baseline evaluation while the class is computed using the clinical information in the last evaluation from the patient. Note that this evaluation might be close to the baseline for some patients and years later for others. This approach aims to answer the question: “Will a patient convert to dementia somewhere in the future?”. Besides being the prevalent strategy in the field, it does not deal with the heterogeneity of the MCI population [27].

(2) *Time Windows approach:* Reduces the time span of the FL approach to a specified temporal frame. A cMCI learning example is created whenever the patient is diagnosed with dementia in a follow-up evaluation whose distance from the baseline is less than the predefined time window (Fig. 1, example A). Patients who remain MCI after the time window period lead to a sMCI learning example (Fig. 1, example B). Patients may originate learning examples assigned to different classes depending on the time windows width. A given patient may be sMCI in a smaller window and originate a converting learning example in a larger window. This is actually what mimics real world situations: a clinician assigns the most likely prognostic for a given patient and this prognostic may change in a later follow-up assessment. We note, however, that not considering these cases would be incoherent as there is no guarantee that a stable MCI would never convert to dementia. In this context, the prognostic refers to a specific time windows and may change if the window changes. Some cases must be disregarded per time window, due to lack of temporal information. For instance, if in the last evaluation the patient remains MCI, but the distance between evaluations is shorter than the time window, he/she is discarded since we cannot guarantee that this patient will not convert until the end of the considered time window (Fig. 1, example C). Similarly, if the patient is diagnosed with dementia in an evaluation outside the window, we cannot guarantee that he/she had already converted within the predefined time window (Fig. 1, example D).

In this scenario, the proposed Time Windows approach reduces the heterogeneity in MCI population caused by the slow cognitive decline characteristic in dementia. As a result, we foresee more accurate prognostic models, as classifiers learn from a set of patients with similar disease progression patterns. In addition, we highlight the clinical relevance of this approach, which allow clinicians to timely adequate treatments to patients and schedule appointments at the hospital.

#### Learning the prognostic model

A prognostic model is trained for each time window and FL dataset following a grid-search strategy, where a set of classifiers and respective parameters, as well as preprocessing options, are tuned (Fig. 2, Learning the model). A cross-validation (CV) procedure is repeated with fold randomization for each classification experiment in order to access model generalization. A grid search is performed to find the optimal set of parameters per classifier. The best parameters are those that achieve the best average on a given evaluation metric across the cross-validations results. The proposed supervised learning approach using time windows may be used with any classifier, preprocessing options and/or types of data.

In this study, we tested the approach with the settings described below and using the cohort of patients recruited in Lisbon (CV set). A 5-fold cross-validation procedure was repeated 10 times with fold randomization for each classification experiment. In order to access the robustness of our hypothesis, we used classifiers that rely on different approaches to the classification problem: Naïve Bayes classifier (NB), Decision Tree (DT) with J48 algorithm as well as Random Forest (RF), Gaussian (SVM RBF) and Polynomial-kernel (SVM Poly) Support Vector Machines (SVMs) using SMO implementation, k-Nearest Neighbor classifier (with IBK implementation, kNN), and Logistic Regression (LR). Table 2 shows the parameters and corresponding ranges tested for each classifier. The grid search criterion was the maximization of the value of the Area Under the ROC Curve [41], as this metric is widely used in binary classification and is appropriate to deal with class imbalance. For simplicity, this metric is referred to as AUC throughout the text. The sensitivity (proportion of actual converting patients (cMCI) which are correctly classified) and specificity (proportion of non-converting patients (SMCI) which are correctly identified) evaluation metrics are also reported.

**Table 2:** Set of parameters and corresponding ranges tested for each classifier within the grid search scheme

*Classifier*	*Parameters and respective range*
NB	*Gaussian or Supervised Discrimination or Kernel*
DT	*Confidence* ∈ [0.05,0.5]
SVM RBF	*Complexity* ∈ [10^−1^, 10^1^] and *γ* ∈ [10^−2^, 10^2^]
SVM Poly	*Complexity* ∈ [10^−1^, 10^1^] and *Degree* ∈ {1, 2, 3}
kNN	#*Neighbors* ∈ [1, 11]
RF	#*Iterations* ∈ [5, 30]
LR	*Ridge* ∈ [10^−9^, 10^−6^]

Note: DT: Decision Tree classifier, kNN: k-nearest neighbor classifier, SVM Poly: polynomial-kernel Support Vector Machines, SVM RB: Gaussian-kernel Support Vector Machines, NB: Naïve Bayes classifier, LR: Logistic Regression and RF: Random Forest

Since the use of preprocessing techniques to deal with a large number of (possibly irrelevant) features, missing values or imbalanced classes may have a significant impact on both classification performance and model simplification and interpretability, the worth of using/not using feature selection and/or dealing with missing values and/or class imbalance should be tested.

In this study, we used Correlation-based (CFS) feature selection [42] to obtain a relevant feature subset. CFS is a filter feature selection (FS) algorithm as the value of a features’ subset is evaluated without taking into account the learning algorithm that is applied afterwards. The method evaluates the worth of a subset of features by resorting to heuristics that consider both the usefulness of individual features to predict the class (in this case, whether the patient converts to dementia (cMCI) or maintains the MCI diagnostic (sMCI)) and the correlation between them.

Although attenuated by feature selection, the problem of missing data still demanded attention and thus missing values were replaced by their mean or mode, whether the attribute was numerical or nominal (Missing Value Imputation, MVI). In addition, class imbalance was tackled with the Synthetic Minority Over-sampling Technique (SMOTE) [43]. SMOTE is an oversampling technique that generates synthetic samples from the minority class by choosing a set of similar instances and perturbing the attributes by a random amount. SMOTE percentages ranged from 0% to the inversion of the class proportions. In order to ensure the validity of the results, all preprocessing techniques (FS, MVI and SMOTE) were only applied to the training data within each cross-validation fold.

The statistical significance of the classification results was evaluated on the averaged AUC across the 10×5-fold CV. The worth of using FS and/or MVI was assessed by the Wilcoxon Signed Rank Test [44], per time window and classifier. Friedman Tests [44] were used to infer whether the results obtained across different classifiers (per time window) have statistical significant differences. Pairwise comparisons (using the Wilcoxon Signed Rank Test) were then performed (with Bonferroni correction for multiple testing) to assess which of those classifiers performed significantly better. To infer whether the predictions made with the Time Windows approach were significantly different from those obtained with the FL approach we used the McNemar’s Test [44]. In this case, the null hypothesis regards the number of correct predictions made by the Time Windows and the FL approaches. We used IBM SPSS Statistics 24 (released version 24.0.0.0) to execute the statistical tests.

#### Validating the prognostic model

An independent validation set (Fig. 2, Validating the model) is used to validate the classification model obtained with the CV set and the subset of features and parameters that best performed in the learning step (Fig. 2, Learning the model). The validation set is independent from the CV set, thus providing a good assessment of model generalization and, simultaneously, a simulation of real world results. In our case, the parameters and preprocessing options were selected exclusively using the Lisbon dataset, which was then used to obtain the prognostic model we validate using the Coimbra dataset.

#### Using the prognostic model in clinical settings

The learned prognostic model can then be used to predict conversion to dementia of new MCI patients. The proposed supervised learning approach using time windows may be integrated in a medical decision support system to be used in clinical settings. This clinical decision support system would predict the most likely prognostic for a new MCI patient based on the past history of a cohort of patients with known prognostics. This prognostic may support the decision of clinicians in real world situations and be useful to adjust treatments and the frequency of the medical appointments.

## Results

We reported the results organized in sections as in the Methods section: 1) Creating learning examples using time windows, 2) Learning the prognostic model and 3) Validating the prognostic model.

### Creating learning examples using time windows

The time windows used in this work are constrained by the follow-up of the cohort under use. In order to avoid skewed class proportion, we were confined to a time span between 1 to 5 years. However, from a clinical point of view, prediction of dementia within 1 year is not very relevant, since by that time, clinicians can easily attain a prognosis. Since many related studies predict 3-year conversion to dementia, including those using ADNI data [8], we decided to consider this window. We thus studied time windows ranging from 2 to 5 years. Table 3 shows the proportion of learning examples in the CV set (patients recruited in Lisbon) and validation set (patients recruited in Coimbra), for each time window and FL datasets. It is expected that as time increases the number of converting patients also increases while the number of patients that remains stable (sMCI) decreases. Datasets built for smaller (2 years) or larger (5 years) time windows have therefore a higher-class imbalance whereas the remaining datasets have minor imbalance. Class imbalance was tackled by using SMOTE in the grid search as aforementioned.

**Table 3:** Details on CV and validation sets for time windows of 2 to 5 years and the First Last approach

	CV set		Validation set	
	*sMCI*	*cMCI*	*sMCI*	*cMCI*
FL approach	377 (62%)	227 (38%)	85 (74%)	30 (26%)
2-Year window	280 (75%)	94 (25%)	53 (80%)	13 (20%)
3-Year window	206 (60%)	137 (40%)	34 (61%)	22 (39%)
4-Year window	146 (47%)	166 (53%)	22 (47%)	25 (53%)
5-Year window	106 (36%)	190 (64%)	10 (28%)	26 (72%)

Note: sMCI- stable MCI; cMCI – converter MCI

### Learning the prognostic model

Regarding the benefit of using missing value imputation, we noticed that Decision Tree, Naïve Bayes and RF classifiers performed better when no imputation was performed (*p* < 0.016,Wilcoxon Signed-Rank Test [44]), considering mean AUC, while kNN benefited from using an imputed version of data (*p* < 0.05,Wilcoxon Signed-Rank Test [44]). We note that in Weka both SVMs (Poly and RBF) and LR already perform MVI internally. Selecting the most relevant set of features achieved significantly better results in most classifiers (kNN, SVM Poly, SVM RBF and LR; *p* < 0.03, Wilcoxon Signed-Rank Test [44]), for all time windows and FL approaches. Although no statistical difference was found for the DT classifier (*p* < 0.269) we decided to proceed with feature selection for the sake of model interpretability. For further analysis, only NB and RF classifiers proceed without FS as their classification performance was significantly improved when using the original set of features (*p* = 0.00,Wilcoxon Signed-Rank Test [44]), considering mean AUC.

The selected subset of features, presented in Table 4, was different for each time window and FL dataset. Particularly, a larger set of features (*n* = 35) was used in the First Last approach when comparing to the Time Windows approach (*n* = 29, in average). From the overall selected features, 14 were commonly chosen throughout all datasets (FL and Time Windows approaches) and 15 within the time windows. This supports the expected differences between datasets comprising patients with distinct times to conversion.

**Table 4:** Subset of selected features for each time window and FL dataset

Features	FL approach	2-Year window	3-Year window	4-Year window	5-Year window
Age	X	X	X	X	X
Age of first symptons	X		X	X	X
*Cancelation Task- A’s time*	X			X	X
*Cancelation Task – A’s total*		X	X		
*Digit Span - Forward*				X	
*Digit Span - Backward*		X	X	X	
*Verbal Paired-Associate Learning – Easy*		X	X	X	
*Verbal Paired-Associate Learning –Difficult*	X	X	X	X	X
*Verbal Paired-Associate Learning – Total*	X	X	X	X	X
*Logical Memory Immediate A free recall*	X	X	X	X	X
*Logical Memory - A Immediate Cued*	X		X	X	
*Word Recall – Free recall*	X	X	X	X	X
*Word Recall –Total*	X		X		
*Logical Memory with Interference-A*	X	X	X		X
*Orientation (Total)*	X	X	X	X	X
*Orientation – Personal*		X	X		
*Orientation – Spatial*					X
*Orientation – Temporal*	X	X	X	X	X
*Orientation- MSQ*	X				
*Verbal Fluency*	X			X	X
Token Orders (total)		X			
*Cube Draw*	X				
*Calculation*		X			
*Interpretation of Proverbs – (Verbal Abstraction)*	X	X	X		
Raven Progressive Matrices	X	X	X	X	X
Trail Making Test (Part B) - time	X				
CVLT A list (1sttrial)				X	X
CVLT A list (3thtrial)			X		
CVLT A list (4thtrial)	X	X			
CVLT A list (five learning trails total)	X	X		X	X
CVLT A list (Total intrusions in 5 recalls)			X		
Blessed Dementia Scale (Total of Part 1 - Daily living activities)	X				
Fi_LM_a	X			X	
Fi_LM_a_m100	X			X	
*Cancelation Task – A’s total* (Z-score)		X	X		
*Digit Span – Forward* (Z-score)		X			X
*Digit Span – Backward* (Z-score)	X		X		
*Digit Span – Total* (Z-score)	X			X	X
*Verbal Paired-Associate Learning* (Z-score)	X	X	X	X	X
*Informatio* (Z-score)	X	X		X	
*Orientation (Total)* (Z-score)	X	X	X	X	X
*Orientation- MSQ* (Z-score)	X	X	X	X	X
*Word Recall –Total* (Z-score)		X			X
*Verbal Fluency* (Z-score)	X	X	X	X	X
*Interpretation of Proverbs – (Verbal Abstraction)* (Z-score)	X		X	X	X
*Raven Progressive Matrices* (Z-score)	X	X	X	X	X
*Cancelation task -Toulouse- Pierón (concentration index)* (Z-score)	X				
*CVLT A list (five learning trails total)* (Z-score)	X		X		
CVLT A list (5sttrial) (Z-score)				X	X
*Logical Memory Immediate A free recall* (Z-score)	X	X	X	X	X
*Logical Memory with Interference-A* (Z-score)		X	X	X	X

The neuropsychological assessment was standardized according to the age and education norms for the Portuguese population and z-scores were calculated

Table 5 shows the results of the stratified 10 × 5-fold CV in the CV set (Lisbon dataset), with the optimized parameters and preprocessing options, for the Time Windows and FL approaches. According to the results, using the Time Windows approach proved to be advantageous over the FL approach (*p* < 0.05, McNemar’s Test [44]). Superior results (in terms of AUC) were reached for the Time Windows approach in all classification experiments and across all classifiers, showing that the conclusions are not dependent on a particular classifier. Sensitivity, which reflects the ability to predict conversion cases, reached better performances within the Time Windows approach, even in the 2-years windows, which has a marked class imbalance. We note that since sensitivity and specificity are sensitive to the number of examples labelled as cMCI and sMCI, respectively, and due to the class imbalance, we expected an increase on the sensitivity and a decrease on the specificity with the widening of the temporal window. Despite this tendency was in general verified exceptions occurred. In the 5-years windows, for instance, some classifiers (DT, kNN, NB and LR) outperformed the specificity reached with the same classifiers on both the 2-years window and FL datasets (where sMCI is the class in majority). In fact, the highest specificity values obtained with the FL approach were achieved at the cost of much lower sensitivity values. The results corroborate the advocated idea: using groups of homogenized MCI patients regarding the time to conversion, and therefore at similar stages of the disease, leads to better performance of the prognostic models.

**Table 5:** Results of stratified 10 × 5-fold cross validation with the CV set (patients recruited in Lisbon, Table 3), under the Time Windows and the First Last approaches

	AUC					Sensitivity					Specificity				
	*FL*	*2Y*	*3Y*	*4Y*	*5Y*	*FL*	*2Y*	*3Y*	*4Y*	*5Y*	*FL*	*2Y*	*3Y*	*4Y*	*5Y*
DT	*0.65 ± 0.02*	***0.71 ± 0.04***	***0.75 ± 0.01***	***0.78 ± 0.02***	***0.79 ± 0.02***	*0.59 ± 0.03*	***0.65 ± 0.05***	***0.75 ± 0.04***	***0.71 ± 0.04***	***0.77 ± 0.02***	*0.68 ± 0.02*	***0.73 ± 0.04***	***0.69 ± 0.03***	***0.77 ± 0.03***	***0.75 ± 0.02***
kNN	*0.67 ± 0.01*	***0.77 ± 0.01***	***0.82 ± 0.01***	***0.83 ± 0.01***	***0.84 ± 0.01***	*0.60 ± 0.02*	***0.70 ± 0.03***	***0.87 ± 0.01***	***0.69 ± 0.03***	***0.83 ± 0.01***	*0.65 ± 0.01*	***0.71 ± 0.01***	*0.61 ± 0.01*	***0.81 ± 0.02***	***0.72 ± 0.03***
SVM Poly	*0.63 ± 0.01*	***0.70 ± 0.01***	***0.76 ± 0.01***	***0.79 ± 0.01***	***0.80 ± 0.01***	*0.43 ± 0.02*	***0.55 ± 0.02***	***0.71 ± 0.01***	***0.81 ± 0.02***	***0.86 ± 0.01***	*0.83 ± 0.01*	***0.84 ± 0.01***	*0.81 ± 0.01*	*0.77 ± 0.01*	*0.75 ± 0.02*
SVM RBF	*0.63 ± 0.01*	***0.64 ± 0.01***	***0.76 ± 0.01***	***0.79 ± 0.01***	***0.80 ± 0.02***	*0.40 ± 0.02*	*0.35 ± 0.02*	***0.72 ± 0.02***	***0.80 ± 0.02***	***0.89 ± 0.01***	*0.86 ± 0.01*	***0.93 ± 0.01***	*0.81 ± 0.01*	*0.78 ± 0.02*	*0.71 ± 0.03*
NB	*0.74 ± 0.00*	***0.82 ± 0.01***	***0.86 ± 0.00***	***0.87 ± 0.01***	***0.88 ± 0.00***	*0.64 ± 0.02*	***0.66 ± 0.01***	***0.75 ± 0.02***	***0.82 ± 0.01***	***0.88 ± 0.01***	*0.71 ± 0.02*	***0.82 ± 0.01***	***0.79 ± 0.01***	***0.78 ± 0.01***	***0.71 ± 0.01***
LR	*0.72 ± 0.01*	***0.79 ± 0.01***	***0.84 ± 0.01***	***0.84 ± 0.01***	***0.85 ± 0.01***	*0.47 ± 0.01*	***0.77 ± 0.02***	***0.85 ± 0.03***	***0.74 ± 0.01***	***0.78 ± 0.01***	*0.80 ± 0.01*	*0.66 ± 0.01*	*0.68 ± 0.02*	***0.81 ± 0.02***	*0.78 ± 0.02*
RF	*0.72 ± 0.01*	***0.79 ± 0.01***	***0.85 ± 0.01***	***0.86 ± 0.01***	***0.87 ± 0.01***	*0.59 ± 0.03*	*0.53 ± 0.04*	***0.75 ± 0.01***	***0.75 ± 0.01***	***0.87 ± 0.01***	*0.71 ± 0.02*	***0.86 ± 0.01***	***0.77 ± 0.02***	***0.81 ± 0.01***	*0.70 ± 0.02*

Note: DT: Decision Tree classifier, kNN: k-Nearest Neighbor classifier, SVM Poly: polynomial-kernel Support Vector Machines, SVM RB: Gaussian-kernel Support Vector Machines, NB: Naïve Bayes classifier, LR: Logistic Regression and RF: Random Forest

The results were highlighted in bold whenever Time Windows approach outperformed the FL approach. cMCI represents the positive class

Within the Time Windows approach, the best results were achieved for larger time windows, namely the 4 and 5-years windows, for all classifiers. Although the highest AUC is consecutively obtained with the 5-years window it might be worth using the 4-years window, since higher values of specificity are obtained without compromising the sensitivity. This may be justified by the inexistence of class imbalance on the 4-years window dataset.

#### Best prognostic model

**Fig. 4:**
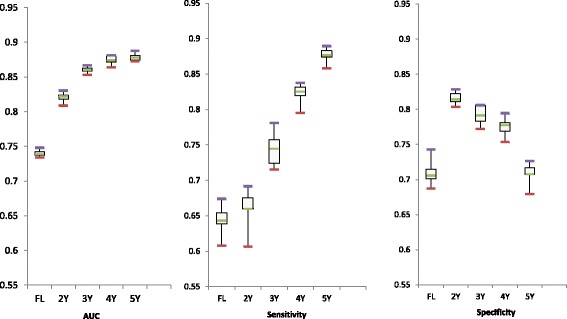
Results obtained with Naïve Bayes, the best classifier for the Time Windows and the First Last approaches, as assessed by the AUC values within a grid search scheme, under 10 × 5-fold cross validation (using the CV set)

The AUC values were statistically different (*p* = 0.00) across classifiers as assessed by the Friedman Test [44]. Therefore, we selected the classifier (with optimized parameters) that gave the best prognostic model to use in further analysis. Following an analysis of pairwise comparisons (with significance values corrected for multiple testing), we concluded that Naïve Bayes was significantly better than the remaining classifiers (for the Time Windows and FL approaches; *p* < 0.002,Wilcoxon Signed-Rank Test [44]). NB is a simple probabilistic classifier, yet robust to class imbalance [45], which has the advantage of returning a numerical confidence of the results, that in turn, can be used as a risk measure by the clinicians. Figure 4 shows the performance obtained with the Naïve Bayes using the CV set.

Once more, we noticed the superiority of the results when using the Time Windows approach instead of the FL approach (*p* < 0.039, McNemar’s Test [44]) and, within those, when larger time windows were used. The FL approach had the lowest performance in all evaluation metrics, with an absolute mean difference of 0.14 (0.14), 0.18 (0.23) and 0.07 (0.001) when comparing to our best results 5 (and 4)-years window in the AUC, sensitivity and specificity, respectively.

Comparing the time windows, we may observe an increase in the AUC as the time window grow, suggesting that the larger the window the more reliable the prognostic model is. However, the drop in the specificity values, mainly observed in the 5-years window, requires attention. As aforementioned, it may be worth using the 4-years window, which despite having lower values of AUC and sensitivity has superior specificity values. The best outcome was then achieved for the 4 and 5-years windows approach (AUC: 0.87 ± 0.01/0.88 ± 0.00, sensitivity: 0.82 ± 0.01/0.88 ± 0.01, specificity: 0.78 ± 0.01/0.71 ± 0.01; 4/5-years windows). To evaluate the impact of patients who converted between 4 and 5 years regarding the other patients, we inspected how many of these patients had a correct prognostic prediction. 20 (average of the 10×5 CV) out of the 24 converting patients were correctly classified as such. This shows the ability of the Time Windows approach to predict conversion as earlier as 5-years before dementia is present.

Despite the class imbalance of the 5-years window dataset (Table 3), it performed better than similarly imbalanced datasets (for instance, the 3-year window). This lead us to the idea that learning the disease patterns of converter MCI is trickiest than learning the patterns of patients who remain stable (sMCI). This is suggested by the smaller fluctuations in the specificity values across distinct times windows, when compared with the sensitivity values, which had greater fluctuations.

### Validating the prognostic model

Table 6 reports the results of the best prognostic model (Naïve Bayes, subsection “Best prognostic model”) derived during the train phase, using the validation set (Fig. 2, Validating the model). We recall that these results are not used to choose the best classifier or parameters, which was done beforehand (Fig. 2, Learning the model). At this step, the best prognostic model was evaluated in an independently (validation) set, for each time window and FL datasets. Comparing the results of Tables 5 and 6, we may observe that most of the conclusions drawn for the CV set are also valid for the validation set. Although the overall results were slightly lower in the validation set, we notice that the Time Windows approach performed better than the FL approach, achieving superior AUC values. Having a lower performance on the validation set corroborates our expectations as we are using an independent set, unbiased from the preprocessing and parameters-tuning procedure. The best outcome was also the one attained with the 5-years window approach. Conversely to what happened in the CV set using the NB classifier, the sensitivity of the 4 and 5-years windows was lower than the respective specificity values. This showed some weakness of the proposed prognostic model in identifying converting MCI patients, in this study cohort. In general the results were good with AUC values above 0.72 for the Time Windows approach suggesting that model overfitting is reduced as aimed by using 10 × 5*-*fold CV to learn and tune the models. The effect of class imbalance (while training the models with the CV set) was not critical in the validation set. Indeed, acceptable values of sensitivity and specificity were attained for the 2-years window (0.69 and 0.66 in the validation and CV set, respectively) and for the 5-years window (0.70 and 0.71 in the validation and CV set, respectively), correspondingly.

**Table 6:** Results of the best prognostic model using the independent validation set (patient recruited in Coimbra, Table 3), for the Time Windows and the First Last approaches

	AUC					Sensitivity					Specificity				
	*FL*	*2Y*	*3Y*	*4Y*	*5Y*	*FL*	*2Y*	*3Y*	*4Y*	*5Y*	*FL*	*2Y*	*3Y*	*4Y*	*5Y*
*Naïve Bayes*	*0.61*	*0.73*	*0.74*	*0.72*	*0.76*	*0.40*	*0.69*	*0.64*	*0.56*	*0.56*	*0.73*	*0.77*	*0.76*	*0.68*	*0.70*

The model was fine-tuned to the CV set (patient recruited in Lisbon, Table 3). cMCI represents the positive class

## Discussion

We proposed a new approach to create learning examples based on time windows, which consists in stratifying the cohort of MCI patients based on their conversion time (converter MCI), or the time that they remained MCI (stable MCI). Then, we evaluated its performance on the prognostic model for MCI-to-dementia conversion by comparing it with the model learned with the FL approach, the prevalent strategy in the field [3, 8, 9, 15, 30, 31]. We showed that, following the FL approach, and thus disregarding the heterogeneity of the population under study caused by the continuous cognitive decline that characterizes this neurodegenerative disease, hampers the discovery of more reliable prognostic models and/or biomarkers. This question had been partially addressed in the literature [33, 34]. Eskildsen et al. [34], homogenized the converter MCI group regarding the time to conversion, using the cortical thickness of anatomical MR images collected at 36, 24, 12 and 6 months before conversion to create the learning examples. Similarly, Cabral et al. [33] created five groups with PDF-PET images collected at 24, 18, 12, 6 and 0 months before conversion. These converting MCI groups, along with the single non-converting MCI group, were fed to machine learning classifiers to perform prognostic. An overall finding was the enhancement of the evaluation metrics with the decrease of the temporal distance to the conversion event. Despite the relevance of this approach, it has been mostly explored with neuroimaging data. We believe that this question is transversal to all biomarker research and thus we performed a similar study using neuropsychological data. To our knowledge, this is the first study using neuropsychological data to predict conversion within a Time Windows approach. We also used the strategy presented in previous works [33, 34] with our data, for sake of comparability. The outcome is shown in appendix [See Additional file 3]. Replicating the methodology pursuit by [33, 34] with our data benefits from a longer follow-up period.

The results support our view about the strengths of predicting conversion to dementia within time windows as this remains true even with different approaches to time windows and data types. Predicting conversion to dementia (cMCI) seems to be the trickiest, suggested by the lower values of sensitivity [33–35]. According to the previous studies, and using neuroimaging data, the accuracy of the prediction improved as the time to conversion from MCI to AD decreased, conversely to our results, where we were able to predict dementia as early as 5-years (AUC: 0.88, specificity: 0.71, sensitivity: 0.88). Our approach, along with neuropsychological data, was thus more successful in the long-term prediction, which we believe to be more useful in the clinical practice and intervention.

One strength of this work was the length of follow-up. We are able to predict conversion to dementia within a long-time span (5 years). Indeed, using neuropsychological data to detect cognitive decline in initial phases of AD has faced significant limitations, due to the short follow-up periods which characterize most cohort studies of conversion to dementia [24, 28, 31, 46]. Our work supports the view that longer follow-ups might be an asset in the study of conversion to dementia, as the best results were achieved with the longest windows used.

Another important point is the sample size. Our cohort has a reasonable size when comparing to similar studies, including those that use data from the industrious ADNI project (study populations of around 200 to 300 patients) [8, 16, 33]. Using a validation set to evaluate how the classification model performs when facing new and unknown data is also to emphasize since it enables to test the model generalization.

We further highlight the use of neuropsychological data to predict dementia. NPTs are relatively inexpensive and non-invasive, can be readily obtained in most clinical settings [23, 24], are required for diagnosis purpose and have proven their value in tracking the cognitive decline in dementia [8]. Still, their predictive power has not been fully exploit, as it has been addressed mostly by classical statistical methods. Indeed, more powerful methods are mainly focused on more complex data, including neuroimaging data and other biological markers. In the present work, we accomplished successful conclusions by using machine learning classifiers with NPTs.

Beyond dealing with the MCI heterogeneity induced by the slow progression nature of dementia, the Time Windows approach takes a step forward in the prognostic research challenge, as it not only predicts whether a MCI patient will evolve to dementia, but also, a time window of conversion.

Some limitations also warrant consideration. The best classifier (and parameters) was chosen based on the AUC values obtained during the grid search. However, it would be preferable to also include the sensitivity and specificity values. It may be worth having smaller AUC values if it allows having equally good values on the remaining classification metrics. The same idea stands for SMOTE which, ideally, should be the lowest possible or not used. Despite many researchers have focused in the MCI-to-dementia conversion problem, comparing these studies is not trivial due to the different data types used, subject inclusion and exclusion criteria, diagnostic criteria for MCI and/or dementia, classification framework and evaluation metrics. The set of common features, as well as the ones that were different across windows, lack a further analysis, from a clinical standpoint, to clarify their clinical relevance. This is however out of the scope of this paper.

## Conclusions

We proposed a supervised learning approach to predict conversion of MCI to dementia based on time windows, following an innovative strategy to build the learning examples and compared it with the commonly used strategy (FL approach). We thus handled the heterogeneity of the MCI cohort by creating different time-homogenous groups regarding their time to conversion (Time Windows approach), when building the learning examples. We studied the effect of disease staging in the performance of the prognostic model by learning different models with different groups of MCI patients, and thus fine-tuning the prognosis regarding the conversion time. The Time Windows approach is more relevant from a clinical point of view, as it provides a temporal interval of conversion thus allowing clinicians to timely adjust treatments and clinical appointments.

Our results corroborated the hypothesized idea, that more reliable prognostic models may be obtained if we handle with the stages of the disease, as Time Windows approach outperformed the First Last approach. Our prognostic model, using neuropsychological data, was able to predict conversion to dementia as early as 5 years before the event.

In the future, we believe that temporal-based classification models may contribute to a better understanding of conversion to dementia and, hopefully, support the decision of clinicians in real world situations. We thus aim to enrich the supervised learning methodology and develop a decision support system to be used in clinical settings: the system would predict, with a given confidence, whether the patient was prone to convert, along with the most likely time window; then, clinicians could use this information to adjust treatments and the frequency of the medical appointments.

Hopefully, this study will encourage researchers to tackle, not only the MCI-to-dementia conversion problem, but also the disease patterns and time to conversion, so we can move to the question on whether a MCI patient will evolve to dementia to the one that predicts the time that will take for this event to happen.

## Additional files

Additional file 1: Table describing the cognitive domains tested by each neuropsychological data of the sample. (DOCX 21 kb)

Additional file 2: Table illustrating the neuropsychological data of the sample. The neuropsychological assessment was standardized according to the age and education norms for the Portuguese population and Z-scores were calculated. (DOCX 26 kb)

Additional file 3: Replication of the methodology proposed in previous works [28, 29] with the data(CCC) used in our study. (DOCX 121 kb)

## Abbreviations

AD: Alzheimer’s disease
AUC: Area under the ROC curve
cMCI: converter MCI
DT: Decision Tree classifier
FL: First Last approach
kNN: k-Nearest Neighbor classifier
LR: Logistic Regression
MCI: Mild Cognitive Impairment
NB: Naïve Bayes classifier
NPTs: Neuropsychological tests
RF: Random Forest
sMCI: stable MCI
SVM Poly: Polynomial-kernel Support Vector Machines
SVM RB: Gaussian-kernel Support Vector Machines
