# Supplementary material for: Predicting progression of mild cognitive impairment to dementia using neuropsychological data: a supervised learning approach using time windows
Source: BMC Med Inform Decis Mak. 2017 Jul 19;17:110. doi: 10.1186/s12911-017-0497-2 (PMC5517828; doi:10.1186/s12911-017-0497-2)
Supplement: Supplementary file 1 — Table describing the cognitive domains tested by each neuropsychological data of the sample. (DOCX 21 kb) [file 12911_2017_497_MOESM1_ESM.docx]

**Table S1.** Description of the neuropsychological tests performed in the Cognitive Complaints Cohort (CCC).

| Cognitive domain | Neuropsychological test | | Feature name | Description |
| --- | --- | --- | --- | --- |
| Global cognitive status | Mini-Mental State Examination (MMSE) | | MMSE_Total | Total score corresponds to the sum of correct answers for five areas of cognitive function: orientation, registration, attention and calculation, recall, and language |
| Motor speed, Sustained focus, alternating attention, visuospatial abilities and cognitive flexibility | Toulouse-Piéron Test | | Cancelation task -Toulouse- Pierón (work efficiency) | Total of symbols assigned correctly within 10 minutes minus omissions and errors (work-efficiency) |
|  |  |  | Cancelation task -Toulouse- Pierón (concentration index) | Concentration index stands for the omissions and errors divided by the correct answers multiply by 100 |
|  | Trail Making Test | | Trail Making Test (Part A) – time | Time in seconds for the completion of part A (connecting numbers 1-25 in ascending order) |
|  |  |  | Trail Making Test (Part B) - time | Time in seconds for the completion of part B (connecting alternating between numbers and letters in ascending order 1-13; A-M) |
|  | Single Letter Cancellation (subtest of Lisbon Battery for the Assessment of Dementia – BLAD) | | Cancelation Task – A’s cuts | Number of letters assigned correctly |
|  |  |  | Cancelation Task- A’s time | Elapsed time for the task |
|  |  |  | Cancelation Task – A’s total | Total score corresponds to the number of letters assigned divided by time multiplied by 10 |
| Personal, temporal and spatial orientation | Orientation (subtest of Lisbon Battery for the Assessment of Dementia – BLAD) | | Orientation (Total) | Total of correct answers for personal, temporal and spatial orientation |
|  |  |  | Orientation – Personal | Total of correct answer for personal orientation |
|  |  |  | Orientation – Spatial | Total of correct answer for spatial orientation |
|  |  |  | Orientation – Temporal | Total of correct answer for temporal orientation |
| Executive control; verbal and non-verbal reasoning; visuoperceptual skills, visuospatial/praxis abilities and semantic knowledge | Initiatives  (subtests of Lisbon Battery for the Assessment of Dementia – BLAD) | Category Fluency | Verbal Fluency | Total of unique correct words from the category specified (food items) |
|  |  | Motor Initiative | Motor Initiative | Total of alternating patterns of motor activity correctly executed |
|  |  | Graphomotor Initiative | Graphomotor Initiative | Total of visual (graphic shapes) alternating patterns correctly reproduced |
|  | Raven's Coloured Progressive Matrices (subtest of Lisbon Battery for the Assessment of Dementia – BLAD) | | Raven Progressive Matrices | Total of items correctly identified to complete the visual patterns (Set Ab) |
|  | Proverbs Interpretation (subtest of Lisbon Battery for the Assessment of Dementia – BLAD) | | Interpretation of Proverbs –  (Verbal Abstraction) | Total score stands for the level of abstraction in the correct interpretation of three proverbs |
|  | Clock Drawing (subtest of Lisbon Battery for the Assessment of Dementia – BLAD) | | Clock Draw | Total score for the free drawning of a clock |
| Visuoconstructive abilities | Cube (subtest of Lisbon Battery for the Assessment of Dementia – BLAD) | | Cube Draw | Total score for the copy of a 3D cube figure |
| Language and calculation | Simple command comprehension (subtest of Lisbon Battery for the Assessment of Dementia – BLAD) | | Orders Compreenshion | Total of correct answers to four simple verbal commands |
|  | Object Identification (subtest of Lisbon Battery for the Assessment of Dementia – BLAD) | | Object Identification | Total of objects identified correctly |
|  | Complex command comprehension (subtest of Lisbon Battery for the Assessment of Dementia – BLAD) | | Token Test, colours | Total for the correct naming of four colours |
|  |  |  | Token Test, orders | Total score for the short version of Token test (verbal complex and nonredundant commands to perform with different sizes of circles and squares) |
|  | Object naming (subtest of Lisbon Battery for the Assessment of Dementia – BLAD) | | Naming | Total of objects correctly named |
|  | Spontaneous Writing (subtest of Lisbon Battery for the Assessment of Dementia – BLAD) | | Writing | Total score for the spontaneous writing of a phrase |
|  | Written Arithmetic (subtest of Lisbon Battery for the Assessment of Dementia – BLAD) | | Calculation | Total score for the correct written calculation |
| Verbal episodic memory and learning | Logical Memory (subtest of Lisbon Battery for the Assessment of Dementia – BLAD) | | Logical Memory Immediate A free recall | Total score for immediate recall of prose (story A) |
|  |  |  | Logical Memory - A Immediate Cued | Total score for immediate cued recall of Prose (Story A) |
|  |  |  | Logical Memory with Interference-A | Total score for delayed recall of prose (story A) |
|  |  |  | Logical Memory with Interference-A Cued | Total score for delayed cued recall of Prose (Story A) |
|  | Word Recall with Interference (subtest of Lisbon Battery for the Assessment of Dementia – BLAD) | | Word Recall – Free recall | Total score for the words free recalled |
|  |  |  | Word Recall – Cued | Total score for the words only recalled with cues |
|  |  |  | Word Recall – Recognition | Total score for the words not recalled but recognized |
|  |  |  | Word Recall –Total | Total score corresponds to the sum of free, cued and recognition |
|  | Verbal Paired-Associate Learning (subtest of Lisbon Battery for the Assessment of Dementia – BLAD) | | Verbal Paired-Associate Learning – Easy | Total of related pairs correctly recalled (first word of the pair is given) |
|  |  |  | Verbal Paired-Associate Learning –Difficult | Total of unrelated pairs correctly recalled (first word of the pair is given) |
|  |  |  | Verbal Paired-Associate Learning – Total | Total score corresponds to the sum of related pairs divided by two plus the sum of unrelated pairs correctly recalled |
|  | California Verbal Learning Test | | CVLT A list (1st trial) | First trial total from free recall of list A |
|  |  |  | CVLT A list (five learning trails total) | Total from trials 1-5 to assess verbal learning abilities |
|  |  |  | CVLT test B list | Total from free recall of list B |
|  |  |  | CVLT A list  (short delayed recall, spontaneous) | Total from short-term delayed recall of list A (following recall of list B) |
|  |  |  | CVLT A list (short delayed recall, semantic cues) | Total from cued short-term delayed recall of list A |
|  |  |  | CVLT A list (long delayed recall) | Total from long-term delayed recall of list A (20 minutes later) |
|  |  |  | CVLT A list  (long delayed recall – recognition) | Total of recognition hits (list A) |
| Working memory | Digit Span (subtest of Lisbon Battery for the Assessment of Dementia – BLAD) | | Digit Span - Forward | Total correct forward repetition of digit series |
|  |  |  | Digit Span - Backward | Total correct backward repetition of digit series |
|  |  |  | Digit Span - Total | Total score of digit span (sum of digit span forward and backward) |
| Visual episodic memory | Visual Memory (subtest of Lisbon Battery for the Assessment of Dementia – BLAD) | | Visual Memory (WMS – B image) | Total score for visual memory reproduction of figure B from the Wechsler Memory Scale (WMS) |
| Remote memory | Information (subtest of Lisbon Battery for the Assessment of Dementia – BLAD) | | Information | Total of correctly retrieved general factual information |
| Depressive symptoms | Geriatric Depression Scale | | GDS | Total for the presence of depressive symptoms |
| Memory decline symptoms | Subjective Memory Complaints Questionnaire | | Subjective Memory Complaints | Total for the presence of subjective memory complaints |
| Activities of Daily living and behaviour | Blessed Dementia Scale | | Blessed Dementia Scale (Total of Part 1 - Daily living activities) | Total score for daily living activities (Part A) |
|  |  |  | Blessed Dementia Scale (Total of Part 2 - Habits) | Total score for change in habits (Part B) |
|  |  |  | Blessed Dementia Scale (Total of Part 3 - Personality) | Total score for change in personality (Part C) |
|  |  |  | Blessed Dementia Scale Total | Sum of Part A, B and C |
