# Supplementary material for: Predicting progression of mild cognitive impairment to dementia using neuropsychological data: a supervised learning approach using time windows
Source: BMC Med Inform Decis Mak. 2017 Jul 19;17:110. doi: 10.1186/s12911-017-0497-2 (PMC5517828; doi:10.1186/s12911-017-0497-2)
Supplement: Supplementary file 2 — Table illustrating the neuropsychological data of the sample. The neuropsychological assessment was standardized according to the age and education norms for the Portuguese population and Z-scores were calculated. (DOCX 26 kb) [file 12911_2017_497_MOESM2_ESM.docx]

Table S2. Neuropsychological data of the sample. The neuropsychological assessment was standardized according to the age and education norms for the Portuguese population and Z-scores were calculated.

|  | sMCI | | | cMCI | | |
| --- | --- | --- | --- | --- | --- | --- |
| **Neuropsychological test** | **M**$\boldsymbol{\pm}$**SD** | **%MV** | **M**$\boldsymbol{\pm}$**SD**  **(z-score)** | **M**$\boldsymbol{\pm}$**SD** | **%MV** | **M**$\boldsymbol{\pm}$**SD**  **(z-score)** |
| BLAD**:**  *Cancelation Task – A’s cuts* | 15.19 ± 1.33 | 8,2% |  | 15.08 ± 1.46 | 8.5% |  |
| *Cancelation Task- A’s time* | 42.80 ± 18.90 | 8,4% |  | 46.27 ± 17.25 | 8.5% |  |
| *Cancelation Task – A’s total* | 4.02 ± 1.88 | 8,4% | 0.25 ± 1.59 | 3.71 ± 1.44 | 8.5% | -0.48±1.26 |
| *Digit Span - Forward* | 4.78 ± 0.75 | 1.1% | -0.23 ± 0.92 | 4.72 ± 0.68 | 0% | -0,38 ± 0.75 |
| *Digit Span - Backward* | 3.56 ± 0.97 | 1.1% | 0.49 ± 1.07 | 3.45 ± 0.93 | 0% | -0.07 ± 0.90 |
| *Digit Span - Total* | 8.34 ± 1.38 | 1.1% | -0.09 ± 0.86 | 8.18 ± 1.35 | 0% | -1.71 ± 0.78 |
| *Verbal Paired-Associate Learning – Easy* | 13.26 ± 3.56 | 1.6% |  | 12.22±- 3.54 | 2.4% |  |
| *Verbal Paired-Associate Learning –Difficult* | 2.95 ± 2.96 | 1.6% |  | 1.67 ±- 2.45 | 2.4% |  |
| *Verbal Paired-Associate Learning – Total* | 9.59 ± 4.25 | 1.1% | -1.24 ± 1.22 | 7.78 ± 3.67 | 2.4% | -1.79 ± 1.03 |
| *Logical Memory Immediate* ***A*** *free recall* | 7.33 ± 4.02 | 0.3% | -1.43 ± 1.06 | 5.44 ± 3.72 | 0.8% | -1.91 ± 0.99 |
| *Logical Memory -* ***A*** *Immediate Cued* | 10.33 ± 4.09 | 32.2% |  | 8.53 ± 4.00 | 20.6% |  |
| *Word Recall – Free recall* | 3.60 ± 3.07 | 5.8% |  | 2.25 ± 2.70 | 4.5% |  |
| *Word Recall – Cued* | 3.76 ± 1.89 | 5.8% |  | 4.08 ± 1.83 | 4.5% |  |
| *Word Recall – Recognition* | 1.33 ± 1.08 | 5.8% |  | 1.44 ±- .93 | 4.5% |  |
| *Word Recall –Total* | 8.66 ± 2.35 | 5.8% | -1,27 ± 1.41 | 7.77 ± 2.34 | 4.5% | -1.72 ± 1.44 |
| *Information* | 17.31 ± 2.92 | 13.7% | -0.23 ± 1.21 | 16.84 ± 3.64 | 12.6% | -0.46 ± 1.48 |
| *Visual Memory (WMS – B image)* | 2.11 ± 1.66 | 70.2% |  | 1.23 ± 1.29 | 70.4% |  |
| *Logical Memory with Interference-A* | 5.51 ± 4.26 | 6.1% | -1.34 ± 0.99 | 3.59 ± 3.85 | 10.1% | -1.72 ± 0.90 |
| *Logical Memory with Interference-A Cued* | 7.62 ± 4.50 | 54.1% |  | 6.11 ± 4.24 | 53.4% |  |
| *Orientation (Total)* | 13.96 ± 1.47 | 1.8% | -24.1 ± 8.74 | 12.71 ± 2.25 | 2.8% | -26.5 ± 10.38 |
| *Orientation – Personal* | 4.94 ± 0.25 | 1.8% |  | 4.80 ± 0.57 | 2.8% |  |
| *Orientation – Spatial* | 2.96 ± 0.23 | 1.8% |  | 2.92 ± 0.31 | 2.8% |  |
| *Orientation – Temporal* | 6.03 ± 1.34 | 1.8% |  | 5.01 ± 1.86 | 2.8% |  |
| *Verbal Fluency* | 14.99 ± 4.80 | 0.3% | -0,32 ± 1.62 | 13.04 ± 4.23 | 0.4% | -0.93 ± 1.44 |
| *Motor Initiative* | 2.68 ± .068 | 2.6% | -0.33 ± 1.89 | 2.57 ± 0.78 | 1.6% | -0.47 ± 1.81 |
| *Graphomotor Initiative* | 1.70± 0.51 | 10.6% | 0.02 ± 0.98 | 1.64 ± 0.53 | 8.9% | -0.11 ± 1.03 |
| *Identification* | 5.00 ± 0.00 | 6.1% | -0.17 ± 0.29 | 5,00 ± 0.00 | 13% |  |
| *Orders Compreenshion* | 3.99 ± 0.11 | 5.3% |  | 4 ± 0.00 | 12% |  |
| *Naming* | 6.94 ± 0.54 | 29.6% |  | 6.95 ± 0.49 | 33.6% |  |
| *Writing* | 1.96 ± 0.23 | 9.8% |  | 1.96 ± 0.19 | 9.3% |  |
| *Token Test Colours* | 4 ± 0.00 | 18.2% |  | 4 ± 0.00 | 21.5% |  |
| *Token Test, orders* | 15.15 ± 3.34 | 18.2% | -0,43 ± 1.74 | 14 ± 0.86 | 21.5% | -0.40 ± 1.57 |
| *Clock Draw* | 2.14 ± 0.93 | 4% | 0.43 ± 0.98 | 2.06 ± 0.19 | 19.4% | 0.29 ± 1.18 |
| *Cube Draw* | 2.61 ± 0.63 | 16.9% | 0.15 ± 1.74 | 2.53 ± 0.69 | 3.2% | 0.05 ± 1.33 |
| *Calculation* | 12.44 ± 2.67 | 11.6% | -0.06 ± 1.25 | 12.08 ± 2.89 | 19.4% | -0.25 ± 1.35 |
| *Interpretation of Proverbs –*  *(Verbal Abstraction)* | 6.77 ± 1.88 | 0.8% | 0.59 ± 1.33 | 6.34 ± 1.88 | 2% | 0.33 ± 1.15 |
| *Raven Progressive Matrices* | 8.20 ± 2.42 | 5% | -0.16 ± 1.11 | 7.46 ± 2.36 | 8.1% | -0.52 ± 1.14 |
| *MMSE* | 27.13 ± 2.49 | 60.7% |  | 25.33 ± 3.31 | 64.4% |  |
| *Cancelation task -Toulouse- Pierón (work efficiency)* | 121.84 ± 57.23 | 66% | -0.67 ± 1.53 | 82 ± 48.38 | 79.8% | -1.82 ± 1.33 |
| Cancelation task -Toulouse- Pierón (concentration index) | 15.82 ± 16.31 | 66% | 0.60 ± 2.32 | 36.39 ± 83.05 | 79.8% | 3.45 ± 11.44 |
| Trail Making Test (Part A) – time | 74.25 ± 39.02 | 49.9% | 0.96 ± 1.94 | 87.06 ± 51.39 | 55.9% | 1.45 ± 1.76 |
| Trail Making Test (Part B) - time | 180.63 ± 88.53 | 49.9% | 1.21 ± 1.96 | 204.3 ±104.07 | 62.3% | 1.96 ± 2.51 |
| CVLT A list (1^st^trial) | 4.71 ± 1.95 | 48.8% | -1.55 ± 1.55 | 3.89 ± 2.04 | 58.7% | -1.97 ± 1.40 |
| CVLT A list (five learning trails total) | 37.57 ± 10.49 | 48.8% | -2.29 ± 1.56 | 29.88 ± 9.66 | 58.7% | -3.41 ± 1.40 |
| CVLT test B list | 4.57 ± 1.91 | 62.8% | -1.05 ± 1.07 | 4 ± 1.77 | 73.7% | -1.25 ± 0.96 |
| CVLT A list  (short delayed recall, spontaneous) | 6.51 ± 3.34 | 62.5% | -1.80± 1.33 | 4.06 ± 3.09 | 73.3% | -2.82 ± 1.39 |
| CVLT A list (short delayed recall, semantic cues) | 8.87 ± 2.93 | 63.6% | -1.75 ± 1.46 | 6.49 ± 2.32 | 72.9% | -2.96 ± 1.38 |
| CVLT A list (long delayed recall) | 7.02 ± 3.92 | 64.4% | -1.97 ± 1.72 | 3.51 ± 3.33 | 75.3% | -3.58 ± 1.63 |
| CVLT A list  (long delayed recall – recognition) | 13.38 ± 2.54 | 64.9% | -1.20 ± 2.11 | 12.94 ± 2.37 | 74.5% | -1.59 ± 2.12 |
| GDS | 5.46 ± 3.78 | 48.8% |  | 4.83 ± 3.51 | 51.4% |  |
| Subjective Memory Complains | 8.48 ± 3.76 | 68.9% |  | 8.18 ± 0.39 | 72.5% |  |
| Blessed Dementia Scale (Total of Part 1 - Daily living activities) | 1.26 ± 0.86 | 53.8% |  | 1.54 ± 0.92 | 34% |  |
| Blessed Dementia Scale (Total of Part 2 - Habits) | 0.03 ± 0.19 | 53.8% |  | 0.02 ± 0.13 | 34% |  |
| Blessed Dementia Scale (Total of Part 3 - Personality) | 2.09 ± 1.59 | 53.8% |  | 2.16 ± 1.46 | 34% |  |
| Blessed Dementia Scale Total | 3.00 ± 1.99 | 53.8% |  | 3.63 ± 1.96 | 34% |  |

*BLAD: Bateria de Lisboa para a Avaliação de Demências; CVLT: California Verbal Learning test, MMSE: Mini-Mental State Examination GDS: Geriatric Depression Scale.
