# Supplementary material for: Predicting progression of mild cognitive impairment to dementia using neuropsychological data: a supervised learning approach using time windows
Source: BMC Med Inform Decis Mak. 2017 Jul 19;17:110. doi: 10.1186/s12911-017-0497-2 (PMC5517828; doi:10.1186/s12911-017-0497-2)
Supplement: Supplementary file 3 — Replication of the methodology proposed in previous works [28, 29] with the data(CCC) used in our study. (DOCX 121 kb) [file 12911_2017_497_MOESM3_ESM.docx]

Figure S1 illustrates the methodology used in (Cabral, Morgado, Campos Costa, & Silveira, 2015; Eskildsen, Coupé, Fonov, Pruessner, & Collins, 2015) to build the learning examples, based on time windows and neuroimaging data. Patients with MCI are firstly divided in two groups: those who remained stable for at least 36 months (stable MCI) and those who converted to dementia (converter MCI). The sMCI group used only baseline images. In the cMCI group, each scan collected from 6 to 36 months before conversion was used to build a learning example, for a specific time windows (of 6 to 36 months).


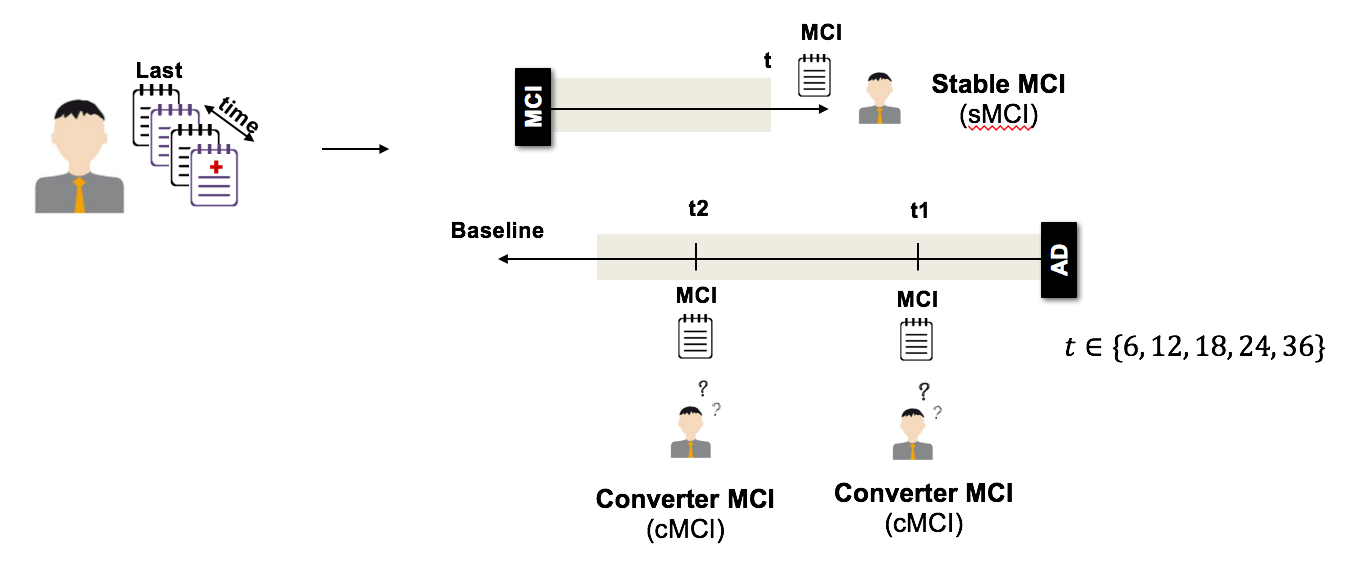


Figure S1- Strategy to construct learning examples based on time windows proposed in (Cabral, Morgado, Campos Costa, & Silveira, 2015; Eskildsen, Coupé, Fonov, Pruessner, & Collins, 2015).

Table S3 presents the proportion of learning examples obtained with the Cognitive Complaints Cohort (patients cohort used in this work) and the strategy described in Figure S1.

Table S3. CV set composition, following the strategy to create learning examples proposed in (Cabral, Morgado, Campos Costa, & Silveira, 2015; Eskildsen, Coupé, Fonov, Pruessner, & Collins, 2015).

|  | CV set | |
| --- | --- | --- |
|  | *sMCI* | *cMCI* |
| FL approach | 320 (62%) | 196 (38%) |
| 1-year window | 67 (45%) | 81 (55%) |
| 2-year window | 67 (72%) | 51 (43%) |
| 3-year window | 67 (60%) | 45 (40%) |
| 4-year window | 67 (69%) | 30 (31%) |
| 5-year window | 67 (84%) | 13 (16%) |

Note: sMCI- stable MCI; cMCI – converter MCI

Figure S2 illustrates the results obtained with the Naïve Bayes classifiers, under stratified 10×5-fold CV, for the time windows and FL approaches (Figure 1), using data described in Table 2. The results show that, even using different data types and approaches, the main conclusion remains true: using time-homogeneous MCI groups to learn prognostic models outperforms the First Last approach, where converter MCI patients are pooled.


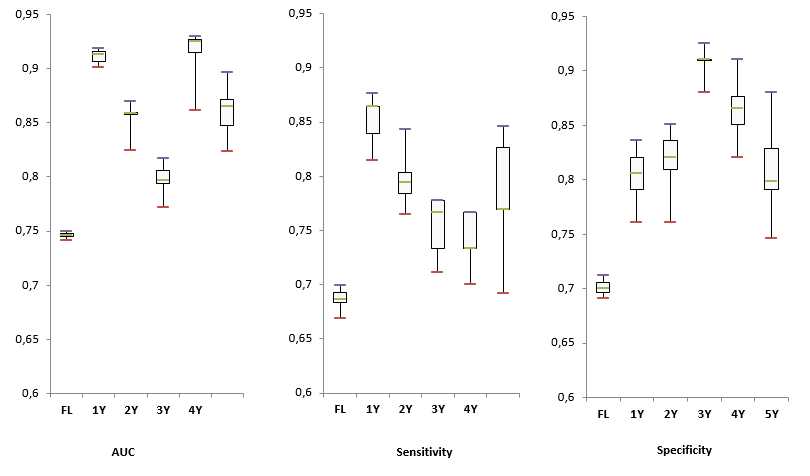


Figure S2- Performance metrics obtained with Naïve Bayes, the best classifier for each time windows and the First Last (FL) datasets, as assessed by the AUC values within a grid search scheme, under 10×5 fold cross validation (using the CV set), when using the learning examples built according to the methodology proposed in (Cabral, Morgado, Campos Costa, & Silveira, 2015; Eskildsen, Coupé, Fonov, Pruessner, & Collins, 2015).
